# The Effects of COVID-19 Pandemic on Public Mental Health: State of Art and Future Perspectives

**DOI:** 10.3390/ijerph20095665

**Published:** 2023-04-27

**Authors:** Matteo Antonio Sacco, Pietrantonio Ricci, Isabella Aquila

**Affiliations:** Institute of Legal Medicine, Department of Medical and Surgical Sciences, University “Magna Graecia” of Catanzaro, 88100 Catanzaro, Italy; matteoantoniosacco@gmail.com (M.A.S.); ricci@unicz.it (P.R.)

The COVID-19 pandemic has radically changed our lives in every way. Since the beginning of the pandemic, more than 760 million cases and nearly 7 million deaths related to the COVID-19 disease have recorded worldwide [1]. The characteristics of the pandemic, both in terms of epidemiological and clinical presentation (since its genesis was not clear at the beginning), and in terms of secondary effects on a global scale, have certainly led to a significant impact on public health and particularly prominent effects upon mental health. Considering this, it is necessary to point out the direct and indirect effects that are associated with the various periods of lockdown and the impact that they have had worldwide [2]. These periods were correlated with unique social phenomena, including the closure of commercial activities, the closure of schools, the prohibition of gatherings in public places and consequently, the requirement to remain at home for extended periods of time. All these phenomena and their consequences regarding public mental health status have been studied comprehensively since 2020. In March 2020, Goyal et al. reported the first case of suicide in India potentially related to the fear of contracting SARS CoV-2 [3]. In April 2020, Gunnell et al. emphasized the criticality of monitoring the epidemiological trend observed in the phenomenon of suicide during the COVID-19 pandemic [4]. The research group coordinated by Prof. Aquila in 2020 also accentuated the importance of monitoring the suicide rate by applying the methodology of psychological autopsy for the first time to a case of suicide related to the pandemic [5]. Furthermore, in 2020, in such a fragile period for the international community, we emphasized that the phenomenon of domestic violence must be monitored, noting the risks associated with being confined at home with one’s cohabitants [6]. This risk could be further intensified in the presence of a violent cohabitant, by virtue of less communication with the outside world and a reduced possibility of the victims being able to access the community and its support structures (through access to schools or in public places). Therefore, since, 2020, the scientific literature has confirmed the critical psychological impact of the COVID-19 pandemic upon mental health.

According to the WHO (World Health Organization), in the first year of the pandemic, there was a noted increase in the prevalence of mental disorders such as anxiety and depression, equal to approximately 25% [7]. In the psychological field, we have considered several potential stress factors, namely:

- Suicide risk related to isolation and financial stress;
- The greater risk of domestic violence in the home environment;
- The removal of young people from community relations within the school and other social fields;
- Burnout within the healthcare community;
- Difficulties in the surveillance and treatment of mental illnesses;
- Reduced access to care for patients suffering from chronic alcohol and/or drug abuse;
- The risk of stigma surrounding COVID-19-positive patients.

Although today, after approximately 3 years, we are witnessing a post-emergency period with a gradual recovery of all activities, scientific interest in the COVID-19 pandemic and its respective effects on mental health remain the subject of numerous studies, especially with respect to the long-term psychological effects of the disease. A recently published study by Xie et al. revealed an increase in the risk of mental illness in subjects who survived the acute phase of the COVID-19 pandemic [8]. In 2023, Hider et al. reported an enhanced risk of mental illness following the COVID-19 pandemic in individuals with rheumatoid arthritis, including anxiety and depression [9].

The evidence that has been offered leads us to further investigate the impact of the COVID-19 pandemic on our psychological states, both in the short and long terms [5]. The purpose of this Special Issue is to provide a platform to academics working within the psychopathological and medical fields and currently undertaking research on the psychiatric effects of COVID-19, with regard to all of the risk factors described. We emphasize the criticality of making progress in scientific research in these fields and within the post-emergency period, in order to carefully monitor the pandemic’s psychopathological effects in the epidemiological and clinical domains, and to apply the appropriate therapeutic and preventative measures to the international community.
